# Analysis of Intensity-Modulated Radiation Therapy (IMRT), Proton and 3D Conformal Radiotherapy (3D-CRT) for Reducing Perioperative Cardiopulmonary Complications in Esophageal Cancer Patients

**DOI:** 10.3390/cancers6042356

**Published:** 2014-12-05

**Authors:** Ted C. Ling, Jerry M. Slater, Prashanth Nookala, Rachel Mifflin, Roger Grove, Anh M. Ly, Baldev Patyal, Jerry D. Slater, Gary Y. Yang

**Affiliations:** Department of Radiation Medicine, Loma Linda University Medical Center, 11234 Anderson Street, A875, Loma Linda, CA 92354, USA; E-Mails: teling@llu.edu (T.C.L.); jeslater@llu.edu (J.M.S.); pnookala@llu.edu (P.N.); rmifflin@llu.edu (R.M.); rgrove@llu.edu (R.G.); amly@llu.edu (A.M.L.); bpatyal@llu.edu (B.P.); jdslater@dominion.llumc.edu (J.D.S.)

**Keywords:** proton, radiotherapy, esophageal, cancer

## Abstract

*Background*. While neoadjuvant concurrent chemoradiotherapy has improved outcomes for esophageal cancer patients, surgical complication rates remain high. The most frequent perioperative complications after trimodality therapy were cardiopulmonary in nature. The radiation modality utilized can be a strong mitigating factor of perioperative complications given the location of the esophagus and its proximity to the heart and lungs. The purpose of this study is to make a dosimetric comparison of Intensity-Modulated Radiation Therapy (IMRT), proton and 3D conformal radiotherapy (3D-CRT) with regard to reducing perioperative cardiopulmonary complications in esophageal cancer patients. *Materials*. Ten patients with esophageal cancer treated between 2010 and 2013 were evaluated in this study. All patients were simulated with contrast-enhanced CT imaging. Separate treatment plans using proton radiotherapy, IMRT, and 3D-CRT modalities were created for each patient. Dose-volume histograms were calculated and analyzed to compare plans between the three modalities. The organs at risk (OAR) being evaluated in this study are the heart, lungs, and spinal cord. To determine statistical significance, ANOVA and two-tailed paired t-tests were performed for all data parameters. *Results*. The proton plans showed decreased dose to various volumes of the heart and lungs in comparison to both the IMRT and 3D-CRT plans. There was no difference between the IMRT and 3D-CRT plans in dose delivered to the lung or heart. This finding was seen consistently across the parameters analyzed in this study. *Conclusions*. In patients receiving radiation therapy for esophageal cancer, proton plans are technically feasible while achieving adequate coverage with lower doses delivered to the lungs and cardiac structures. This may result in decreased cardiopulmonary toxicity and less morbidity to esophageal cancer patients.

## 1. Introduction

Esophageal cancer is a highly aggressive malignancy that accounts for over 400,000 deaths worldwide [1]. Data have demonstrated that neoadjuvant concurrent chemoradiotherapy improves local control and survival compared with surgery alone [2,3,4]. The overall prognosis of esophageal cancer, however, remains poor. It is therefore important to weigh the morbidity of treatment toxicity with the reported benefit of multimodality treatment. Radiotherapy has a clearly defined role in the treatment of esophageal carcinoma but the greatest challenge is to deliver it accurately while minimizing toxicity.

3D conformal radiotherapy (3D-CRT) utilizes X-ray beams that enter and exit the body, creating both entrance and exit doses. As a result, non-targeted organs surrounding the esophagus, namely the heart and lungs, are also exposed to radiation. Pulmonary complications such as radiation pneumonitis and fibrosis are seen in as many as 30% of cases [4]. Recent evidence also suggests that radiation exposure may have a significant impact on postoperative pulmonary complications [5]. The effects of radiotherapy on the heart have been well-documented in patients treated for lymphoma and breast cancer [6]. Radiation therapy has, in fact, been demonstrated as an independent predictor of perioperative cardiopulmonary complications. It was shown to be a major modifiable factor that can mitigate postoperative cardiopulmonary complications [7]. There is, nonetheless, a paucity of data that investigates radiation toxicity of sub-cardiac components. There is very little existing dose-volume histogram data for cardiac structures such as the left ventricle and pericardium, particularly with regard to proton radiotherapy for esophageal cancer. Radiation exposure to the heart has been associated with a higher prevalence of left ventricular ischemia and myocardial perfusion abnormalities [8]. The location of distal and gastroesophageal (GE) junction tumors, however, makes it difficult to reduce heart exposure without compromising tumor coverage.

Improvements in radiation delivery techniques have resulted in methods to improve beam conformity around treatment targets. Intensity-modulated radiation therapy (IMRT) is one such method that utilizes multiple beam angles at varying intensities to escalate dose at the target while sparing surrounding normal tissue from high-dose regions. Proton radiotherapy, another form of radiation treatment, utilizes charged-particle beams. A proton beam deposits most of its energy at a discrete depth within tissue; this deposition is called the “Bragg peak.” The Bragg peak is predictable and can be created to match the exact depth and thickness of the tumor target. The entirety of the beam’s energy is deposited in the target volume, so there is no subsequent exit dose. Some previous dosimetric studies have shown a potential benefit for proton radiotherapy in the treatment of esophageal cancer [9]. To our knowledge, this is the first study to investigate the dosimetric outcomes of sub-cardiac structures in patients receiving proton radiotherapy for esophageal cancer. The purpose of this study was to quantify the dosimetric changes seen in using IMRT, protons or 3D-CRT with consistent planning parameters in patients receiving multimodality therapy for esophageal cancer.

## 2. Experimental

### 2.1. Patient Selection

Ten patients at our institution who underwent radiation treatment for esophageal cancer between 2010 and 2013 were included in our study. The clinical staging (per the American Joint Committee on Cancer, 7th edition) ranged from T_2-3_N_0-1_M_0_ (see Table 1). However, nine of the ten patients had T_3_ disease and seven patients had N_0_ disease. All patients in this study had tumors of the distal esophagus or GE junction.

**Table 1:** Patient characteristics.

Patient	Histology	Tumor Location	TNM Stage	Stage Grouping	Treatment (Gy/fx)	PTV volume (cm^3^)
1	Adenocarcinoma	Distal 2/3 of esophagus	T3 N1 M0	III	50.4/28	1004.47
2	Adenocarcinoma	AEG I	T3 N0 M0	IIA	50.4/28	1174.1
3	Adenocarcinoma	AEG I	T3 N1 M0	III	50.4/28	876.76
4	Adenocarcinoma	AEG II	T3 N1 M0	III	50.4/28	1416.68
5	Adenocarcinoma	Distal 2/3 of esophagus	T2 N0 M0	IIA	50.4/28	666.47
6	Adenocarcinoma	AEG II	T3 N1 M0	III	50.4/28	1866.24
7	Adenocarcinoma	AEG I	T3 N1 M0	III	50.4/28	1509.503
8	Adenocarcinoma	AEG II	T3 N1 M0	III	50.4/28	567.41
9	Adenocarcinoma	Distal 2/3 of esophagus	T3 N1 M0	III	50.4/28	1084.7
10	Adenocarcinoma	Distal 2/3 of esophagus	T3 N0 M0	IIA	50.4/28	813.12

### 2.2. Simulation and Treatment Planning

Patients were simulated in the supine position using intravenous and oral contrast-enhanced CT imaging (GE Lightspeed VCT scanner; GE Healthcare, Little Chalfont, UK) with 2.5-mm slice thickness. All patients were scanned from above the level of the top of the lungs to the iliac crest. All treatment plans were created with the Odyssey 4.8 planning system (Optivus, San Bernardino, CA, USA). Contrast was utilized in each CT scan to delineate the nodes with disease involvement. The gross tumor volume (GTV) consisted of the esophageal tumor and surrounding radiographically involved lymph nodes. The clinical target volume (CTV) was defined as the GTV plus a 1-cm radial expansion and a 3-cm expansion in the superior and inferior directions. The celiac axis was included in the CTV if located within 1 cm of the tumor CTV expansion. The planning target volume (PTV) was generated by expanding the CTV by 10–15 mm, depending on the treatment modality used. Setup uncertainty from respiratory motion and diaphragm movement was accounted for with close attention to target expansions. The planning target volume (PTV) was generated by expanding the CTV by 1.5 cm and 1.2 cm for the 3DCRT and IMRT plans, respectively. All 3DCRT plans are given a 1 cm margin beyond the PTV to the block edge to account for beam penumbra. The lateral penumbra and distal margin of proton plans generated by the treatment planning system were between 1–1.5 cm and based on the beam energy selected. A dose of 50.4 Gy given in 28 fractions was delivered to the PTV. All plans were optimized to allow 90% isodose coverage of at least 95% of the PTV. The primary goal of each plan was first to obtain adequate target coverage. The proton plan beam arrangements consisted of 2 beams at lateral and oblique angles. Lung, heart, and spinal cord were avoided when possible. The median proton beam energy was 250 MeV (range 150–250 MeV) with some minor deviation depending on the distal depth of the target. The depth of the proximal and distal edge helped determine the beam energy selected. Our institution uses a passive scattering beam system, which requires a patient portal-specific collimating aperture to shape the dose to the target field laterally. A range compensator is used to conform the dose to the distal aspect of the target volume. A spread-out Bragg peak to cover the target in the beam direction is achieved with a modulator wheel. All IMRT plans consisted of 6 to 9 non-parallel-opposed 6 MV photon beams delivered with a multi-leaf collimator using a step-and-shoot approach. Critical structure dose was optimized with the inverse planning algorithm. The 3D-CRT plans each consisted of a 4-field box (AP, PA, RL, LL) using 15–24 MV photon beams delivered with either a multi-leaf collimator or custom-cut block. Weighting was adjusted to try to stay within dose constraints of normal tissue being irradiated.

### 2.3. Plan Evaluation and Analysis

Dose-volume histograms (DVH) were calculated and analyzed in order to compare plans from the three different modalities. The organs at risk (OAR) being evaluated in this study are the lung, liver, stomach, heart, and spinal cord. Analysis was performed for the volume of lung receiving 5 Gy (V_5_), 10 Gy (V_10_), 15 Gy (V_15_), 20 Gy (V_20_), 30 Gy (V_30_), 40 Gy (V_40_), 50 Gy (V_50_) as well as the mean lung dose. The stomach V_20_ and V_50_, the dose delivered to 1/3 of the liver (D_1/3_), 2/3 of the liver (D_2/3_), mean liver dose, heart V_25_, V_30_, V_40_, V_50_, mean heart dose and the maximum spinal cord dose were also analyzed. Within the heart, dose-volume data were analyzed for the mean and maximum left anterior descending artery (LAD), left ventricle (LV), and pericardium. All structures of the heart and the lungs were contoured per the validated University of Michigan heart atlas [10,11]. The pericardium was drawn as a “shell” extended 3D outward by 0.5 cm in thickness. The heart volume itself was then subtracted from this expansion. This method has been utilized by several investigators [12]. The target OAR dose constraints used in this study are outlined in the CROSS protocol. Our goal was to keep lung V_20_ < 30%, heart V_40_ < 30%, and liver V_30_ < 60% if possible during treatment planning.

Conformity indices were obtained and analyzed for plans between the three treatment modalities. The homogeneity index (HI), defined as the difference between the maximum and minimum dose to the target volume (D1% and D99%, respectively) divided by the prescription dose [13,14]. Uniformity index (UI) was also used and defined as the ratio of D5% to D95% [15,16]. Both HI and UI were utilized to assess overall plan uniformity per previously established methodology [17]. The conformity index (CI) was defined, per RTOG guidelines, as the volume of the 95% isodose curve divided by the PTV volume. To determine statistical significance, ANOVA and two-tailed paired t-tests were performed for all data parameters. Conformity indices were analyzed with one sample t-tests as well. A *p*-value < 0.05 considered to be statistically significant.

## 3. Results

**Figure 1:**
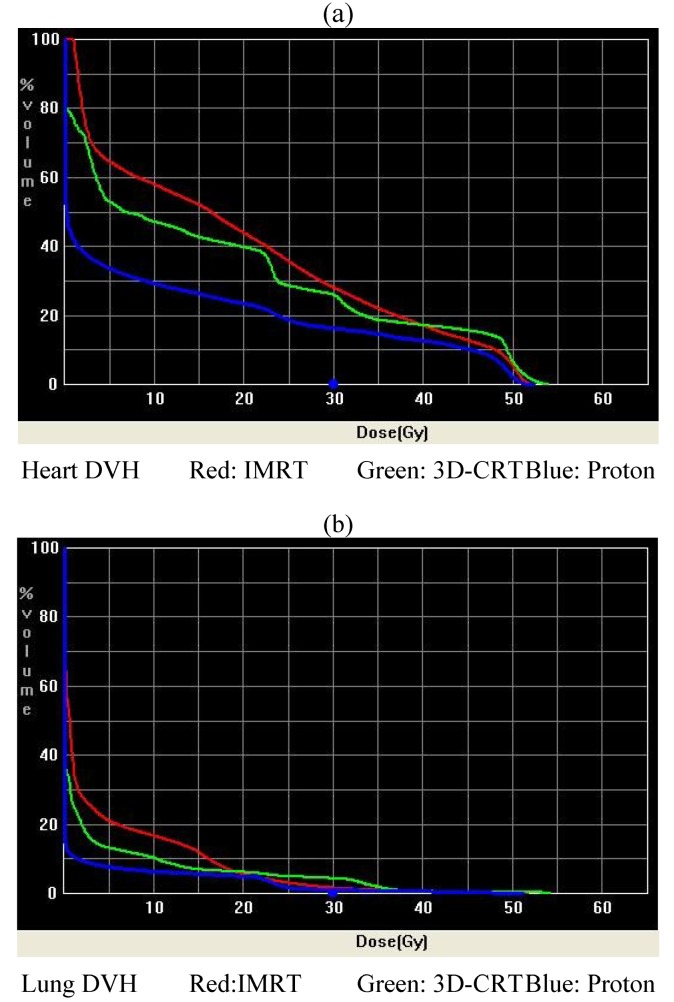
(**a**,**b**) Heart and lung DVHs.

A total of 10 patient scans were utilized for this study. Three treatment plans were created on each scan: proton, IMRT, and 3D-CRT. Table 2 presents dose-volume parameters obtained from these plans. Dose distributions for liver, kidney, and small bowel from two of our study patients are presented in Figure 1. The CTV was encompassed by the 95% isodose line in all cases. At least 95% of the PTV was encompassed by the 90% isodose line. Separate plans were generated and optimized for all 10 study patients. The GTV and CTV were held constant in each patient for each of the three plans.

### 3.1. Protons vs. IMRT

First, we compared dose-volume parameters from IMRT plans with those of the proton plans. The proton plans resulted in lower mean lung dose (6.03 Gy *vs.* 9.45 Gy, *p* = 0.016), lung V_5_ (21.4% *vs.* 46.93%, *p* = 0.001), V_10_ (19.37% *vs.* 37.75%, *p* = 0.003), V_15_ (17.29% *vs.* 27.89%, *p* = 0.009), mean liver dose (3.62 Gy *vs.* 18.13 Gy, *p* = 0.000), liver D_1/3_ (0.20% *vs.* 20.99%, *p* = 0.000), D_2/3_ (0.02 Gy *vs.* 12.44 Gy, *p* = 0.000), and a lower maximum dose to the spinal cord (11.61 Gy *vs.* 36.93 Gy, *p* = 0.000). Also evident is decreased dose to the heart globally. There was a significant reduction in mean heart dose (12.56 Gy *vs.* 28.5 Gy, *p* = 0.000), heart V_25_ (23.71% *vs.* 54.11%, *p* = 0.000), V_30_ (20.85% *vs.* 42.28%, *p* = 0.001), V_40_ (16.21% *vs.* 25.45%, *p* = 0.036), V_50_ (2.8% *vs.* 12%, *p* = 0.008), LAD, LV, and pericardium doses in the proton plans (see Table 2).The proton and IMRT plans provided similar conformity (0.89 *vs.* 1.15, uniformity (1.29 *vs.* 1.11), and homogeneity (0.39 *vs.* 0.16).

**Table 2:** Averaged over 10 patients DVH parameters (±SD) with *p*-values for comparison.

Organ at Risk		Proton Plans	IMRT Plans	3DCRT Plans	*p*-value		
					Proton *vs.* IMRT	Proton *vs.* 3DCRT	IMRT *vs.* 3DCRT
Lung	V5 (%)	21.4 ± 10.3	46.9 ± 17.6	34.1 ± 13.9	0.001	0.032	0.087
	V10 (%)	19.4 ± 8.6	37.8 ± 14.7	29.1 ± 12.7	0.003	0.060	0.178
	V15 (%)	17.3 ± 7.5	27.9 ± 8.8	23.9 ± 11.3	0.009	0.141	0.390
	V20 (%)	15.3 ± 6.5	16.2 ± 5.8	22.1 ± 10.8	0.794	0.114	0.144
	V30 (%)	6.1 ± 2.9	6.6 ± 3.2	9.8 ± 5.1	0.720	0.067	0.113
	V40 (%)	4.3 ± 2.1	3.5 ± 2.0	4.7 ± 2.9	0.391	0.682	0.270
	V50 (%)	1.1 ± 1.0	1.6 ± 1.3	3.3 ± 2.1	0.251	0.008	0.043
	Mean (Gy)	6.0 ± 2.6	9.5 ± 3.2	9.4 ± 4.0	0.016	0.040	0.966
Stomach	V20 (%)	66.8 ± 26.0	87.4 ± 22.9	85.1 ± 25.1	0.076	0.126	0.834
	V50 (%)	29.8 ± 21.7	59.9 ± 28.0	40.0 ± 39.5	0.015	0.484	0.211
Liver	D 1/3 (Gy)	0.2 ± 0.1	21.0 ± 4.7	28.9 ± 7.4	0.001	0.000	0.011
	D 2/3 (Gy)	0.1 ± 0.1	12.4 ± 6.6	11.1 ± 11.8	0.001	0.016	0.754
	Mean (Gy)	3.6 ± 1.8	18.1 ± 4.6	20.3 ± 6.3	0.001	0.001	0.383
Spinal Cord	Dmax (Gy)	11.6 ± 10.0	36.9 ± 3.5	31.2 ± 9.7	0.001	0.001	0.097
Heart	V25 (%)	23.7 ± 7.8	54.1 ± 15.2	56.3 ± 22.3	0.001	0.001	0.802
	V30 (%)	20.9 ± 7.1	42.3 ± 15.3	32.7 ± 9.4	0.001	0.005	0.109
	V40 (%)	16.2 ± 6.4	25.5 ± 11.0	25.8 ± 8.8	0.036	0.012	0.942
	V50 (%)	2.8 ± 2.3	12.0 ± 8.6	20.0 ± 12.6	0.008	0.002	0.118
	Mean (Gy)	12.6 ± 3.9	28.5 ± 5.5	27.5 ± 5.2	0.001	0.001	0.692
LAD	Mean (Gy)	0.4 ± 0.6	17.6 ± 5.8	15.1 ± 8.2	0.001	0.001	0.441
	Max (Gy)	5.4 ± 8.7	31.4 ± 3.6	26.9 ± 4.8	0.001	0.001	0.028
Left Ventricle	Mean (Gy)	13.9 ± 6.6	30.3 ± 5.6	27.3 ± 5.6	0.001	0.001	0.250
	Max (Gy)	51.4 ± 0.9	52.3 ± 0.8	50.6 ± 10.6	0.029	0.808	0.613
Pericardium	Mean (Gy)	13.5 ± 1.8	26.0 ± 5.1	24.8 ± 4.2	0.001	0.001	0.552
	Max (Gy)	52.6 ± 1.3	53.4 ± 0.5	55.3 ± 2.0	0.118	0.003	0.015

### 3.2. Protons vs. 3D-CRT

The next comparison looked at dose-volume parameters between proton plans and 3D-CRT plans. The proton plans delivered a lower mean lung dose (6.03 Gy *vs.* 9.38 Gy, *p* = 0.040), lung V_5_ (21.4% *vs.* 34.12%, *p* = 0.032), lung V_50_ (1.1% *vs.* 3.3%, *p* = 0.008) mean liver dose (3.62 Gy *vs.* 20.34 Gy, *p* = 0.000), liver D_1/3_ (0.20 Gy *vs.* 28.89 Gy, *p* = 0.000), D_2/3_ (0.02 Gy *vs.* 11.08 Gy, *p* = 0.016), and maximum spinal cord dose (11.61 Gy *vs.* 21.23 Gy, *p* = 0.000). Again, the proton plans demonstrated a global reduction in dose delivered to the heart and its components in comparison to the 3D-CRT plans. There was a deceased mean heart dose (12.56 Gy *vs.* 27.53 Gy, *p* = 0.000), heart V_25_ (23.71% *vs.* 56.28%, *p* = 0.001), V_30_ (20.85% *vs.* 32.72%, *p* = 0.005), V_40_ (16.21% *vs.* 25.78%, *p* = 0.012), V_50_ (2.8% *vs.* 20%, *p* = 0.002) LAD, LV, and pericardium (see Table 2). The proton and 3D-CRT plans provided comparable conformity (0.89 *vs.* 1.27), better uniformity (1.29 *vs.* 1.11), and homogeneity (0.39 *vs.* 0.15).

### 3.3. IMRT vs. 3D-CRT

Finally, we compared dose-volume parameters between IMRT plans with 3D-CRT plans. The IMRT plans resulted in a lower liver D_1/3_ (20.99 Gy *vs.* 28.89 Gy, *p* = 0.011), lung V_50_ (1.6% *vs.* 3.3%, *p* = 0.043), max LAD, and max pericardium dose. No statistically significant difference was seen in the other organs at risk. There was also no difference in homogeneity, uniformity, or conformity between the IMRT and 3D-CRT plans.

## 4. Discussion

**Figure 2:**
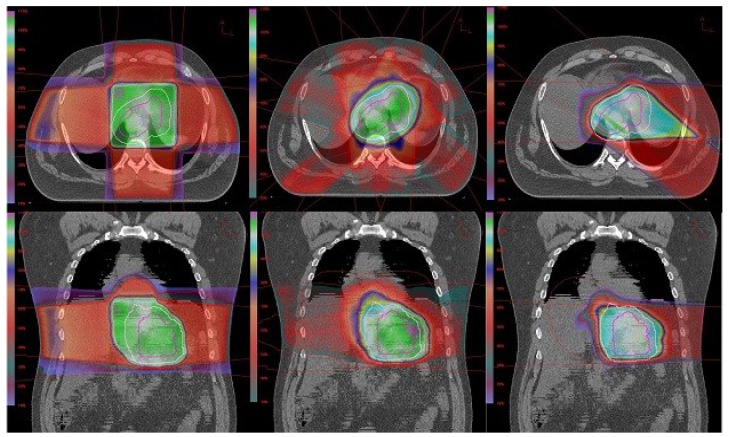
Coronal and transverse images of 3D-CRT plan (left), IMRT plan (middle), proton plan (right).

The results of our study demonstrate a significant tissue-sparing benefit of proton plans over the IMRT and 3D-CRT plans. Target coverage was adequate in each of these treatment planning modalities but the amount of normal tissue irradiated differed between them. Clinically acceptable plans were generated for all 10 patients with each of the three treatment modalities. Target coverage was achieved in each plan despite a significant patient-to-patient variation in target volume size and shape. PTV volumes ranged from 567 cc to 1,510 cc (median 1,045 cc) suggesting that each of these three modalities could provide clinically acceptable treatment plans at least in terms of target coverage. Figure 2 demonstrates beam coverage of the three different modalities in one of the study patients.

The esophagus is closely surrounded by a number of critical organs that stand to be at great risk during radiation therapy. Numerous studies have demonstrated a need for improved treatment planning techniques in esophageal cancer [18,19]. Pulmonary toxicity is a serious side effect associated with radiation therapy. Previous studies have investigated a number of DVH parameters and their predictive value for pulmonary toxicity [20]. In particular, the volumes of lung receiving lower doses, such as V_5_, V_10_, V_15_, and V_20_ are of interest to investigators. Mean lung dose and V_20_ are two of the most commonly implicated DVH parameters that independently predict for pulmonary toxicity [21,22]. Our data show the proton plans delivered a lower dose of radiation to the lungs in comparison to IMRT and 3D-CRT plans. This proton dose reduction is more dramatic in the volumes of lung receiving a low dose. The mean lung dose was also lower in the proton plans. Interestingly, there was very little difference in lung dose between the IMRT and 3D-CRT plans. This may be due in part to the close proximity and large volume of lung surrounding the esophageal tumors. Protons have an inherent dosimetric advantage over photons due to their lack of exit dose, thus resulting in a lower integral dose delivered to the lung. IMRT allows for more conformal dose escalation to the tumor but at the cost of an increased volume receiving a low dose of radiation. Emerging data demonstrate V_5_ as a significant predictor of radiation pneumonitis, so future studies should pay close attention to quantities of volume receiving even very low doses [23]. The proton plans in our study demonstrated a more marked reduction in dose delivery to the V_5_ region, more so than both the V_10_–V_20_ regions. Many esophageal cancer patients undergo chemotherapy concurrently with radiation, followed by surgery. The use of multimodality therapy may require further minimization of the lung volume receiving low doses of radiation. Specifically, there was a significant increase in postoperative pulmonary complications when the V_10_ ≥ 40% [24]. These data highlight the potential benefit protons may offer in reducing volume of lung irradiated by even low doses of ionizing radiation.

An excess risk of heart toxicity has been associated with treatment doses delivered during radiation therapy. Radiation-induced heart disease is a constellation of diseases, including coronary artery disease, pericarditis, heart failure, and pericardial effusion. Traditionally, the entire heart was contoured as a single OAR. Dose was measured as the dose absorbed by the entire volume of the heart irrespective of cardiac structures. More recently, studies indicate that there may be a difference in risk of cardiac toxicity depending on the portion of the heart that is treated [25,26]. Several studies have observed increased rates of radiation-induced heart disease when the heart receives greater than 40 Gy. The V_40_ parameter measured in numerous studies had a strong association with increased rates of heart toxicity [27,28,29]. QUANTEC guidelines indicate increased long-term cardiac mortality associated with V_25_ ≥ 10%. Several studies have looked at heart ejection fraction of esophageal cancer patients in the setting of concurrent chemoradiotherapy. A decrease in both systolic and diastolic left ventricular function has been observed as early as 1 to 3 days after completing concurrent chemoradiotherapy [30]. Myocardial perfusion abnormalities with significant with inferior left ventricular wall ischemia has been noted prominently in distal esophageal cancer. One study utilized gated myocardial perfusion imaging (BMPI) found a higher prevalence of inferior wall ischemia encompassed in radiation isodose lines ≥ 45 Gy [8]. Ejection fraction must be considered in the setting of measured clinical outcomes as a significant decrease in measured dose may not necessarily correlate with clinical outcomes.

The pericardium is another heart structure shown to have dose-dependent radiation toxicity. The underlying mechanism is thought to occur through endothelial cell damage and microvascular injury resulting on a progressive dysfunction to both pericardial and adjacent myocardial soft tissue. The V_30_ parameter has been closely associated with the rate of pericardial effusion. Specifically, a mean pericardial dose of 26.1 Gy and V_30_ > 46% has been associated with significantly increased rates of pericardial effusion [12]. A follow up study confirmed V_30_ as well as V_45_ as predictive values for pericardial effusion for esophageal cancer patients who underwent chemoradiation therapy [31].The proton plans in our study demonstrated a significant reduction in volume of heart irradiated across all parameters. Our data show that the proton plans spare more heart volume than both the IMRT and 3D-CRT plans. This dose reduction is reflected in both the volumes receiving low and high dose as well as the mean heart dose. There no difference in the irradiation volumes between the IMRT and 3D-CRT plans. This was unsurprising given the close proximity of the heart to the GE junction.

Irradiation of the left anterior descending (LAD) artery and left ventricle poses a particular risk to developing coronary artery and ischemic heart disease [32]. The proton plans delivered a significantly lower dose to the LAD and left ventricle in comparison to both the IMRT and 3D-CRT plans. The ability to select particular beam angles in the proton plans is of marked benefit. Again, there was very little difference in dose delivered to the LAD and left ventricle when the IMRT and 3D-CRT plans were compared. The laterality of heart irradiation may also impact the risk of cardiac disease. Many studies showed a higher incidence of cardiac disease after left-sided heart irradiation when compared to irradiation of the right side of the heart [33,34]. Breast cancer data have shown ipsilateral involved-field coronary artery stenosis rates to be higher on the treated side than the contralateral uninvolved side [35]. This phenomenon suggests that certain regions of the heart pose a higher risk for heart disease than others when irradiated. There is little published data discussing the radiation dose tolerance of the coronary arteries and ventricular tissue. Historically, the heart and its subunits have been contoured and treated as a “parallel organ,” meaning a small volume of heart receiving a high radiation dose was considered acceptable. However, any radiation damage to even a small portion of a coronary artery could result in perfusion defects with severe consequences. Some studies suggest treating the certain portions of the heart as a “series organ” instead [36]. In this case, proton plans may offer a higher degree of freedom in beam arrangement, resulting in superior selective sparing of certain heart structures.

Several studies have been done comparing these modalities of treatment focusing on plan optimization and target coverage in esophageal cancer. Zhang *et al*. [9] compared IMRT and proton plans in the treatment of distal esophageal tumors. They demonstrated that their proton plans consistently spared larger volumes of lung as well as reduced mean dose to lung. Isaccson *et al.* [37] compared proton and photon treatment plans for esophageal cancer using a tumor control probability (TCP) and normal tissue complication (NTCP) model. A consistent therapeutic ratio benefit was seen in the proton plans. The radiation modality utilized can be a strong mitigating factor of perioperative complications given the location of the esophagus and its proximity to the heart and lungs. Thus, it is critical to find ways of minimizing radiation toxicity. Martin *et al.* compared several IMRT techniques and found that acceptable OAR doses could be achieved while maintaining adequate target coverage in treating esophageal cancer [38]. More studies must be done to further evaluate dosimetric benefits between these radiotherapy modalities.

Our study revealed that the IMRT and 3D-CRT plans generated relatively large low-dose regions in comparison to proton plans. In our study, there was no significant difference in the volume of the low-dose regions treated by the IMRT and 3D-CRT plans. This was indicated by the lack of a significant difference between the V_5_–V_20_ lung, mean lung dose, and V_25_ heart parameters. In contrast, the proton plans resulted in significantly smaller low-dose regions in comparison to IMRT. The optimal radiotherapy technique for cancer of the distal esophagus and gastroesophageal junction is still unique for each patient. There was consistent overlap between the PTV and OAR, so that no one technique could simultaneously achieve full target coverage while fully respecting OAR constraints. Furthermore, the size and extent of the target volume may preclude the use of certain modalities. Nonetheless, significant conclusions may still be drawn from these generated plans in which full target coverage was obtained with reasonable uniformity and conformity. The uncertainty of the distal range of proton beams and its sensitivity to tissue density changes along their path are potential limitations during proton treatment planning. Therefore, the range errors due to diaphragm motion and stomach gas-filling must be considered in evaluating target coverage and critical structure dose. Great care must be taken in creating adequate target expansions and custom immobilization techniques for each patient in order to account for these uncertainties. Target volume motion during respiration may significantly affect beam selection during the planning process. Currently, our institution utilizes the SpyroDyn’RX (SDX) voluntary breath-hold technique in a phase II trial as a means of active breathing control for more accurate target delineation and treatment delivery.

## 5. Conclusions

There was no difference between the IMRT and 3D-CRT plans in dose delivered to the lung or heart. Target treatment plan conformity was comparable between the three modalities. The proton plans delivered lower heart doses compared to both the IMRT and 3D-CRT plans. The proton plans also resulted in less lung dose in comparison to the IMRT plans. Our data suggests that proton radiotherapy may help improve the therapeutic ratio for patients receiving radiotherapy during multimodality esophageal cancer treatment. Further studies are needed to identify ways to improve radiation therapy techniques in order to minimize risk of cardiac and pulmonary injury in the perioperative setting.
